# Supplementary figures and images for: Medical student research productivity and scholarly impact: A 20-year bibliometric comparison with medical residents
Source: PLoS One. 2026 Feb 23;21(2):e0343160. doi: 10.1371/journal.pone.0343160 (PMC12928410; doi:10.1371/journal.pone.0343160)

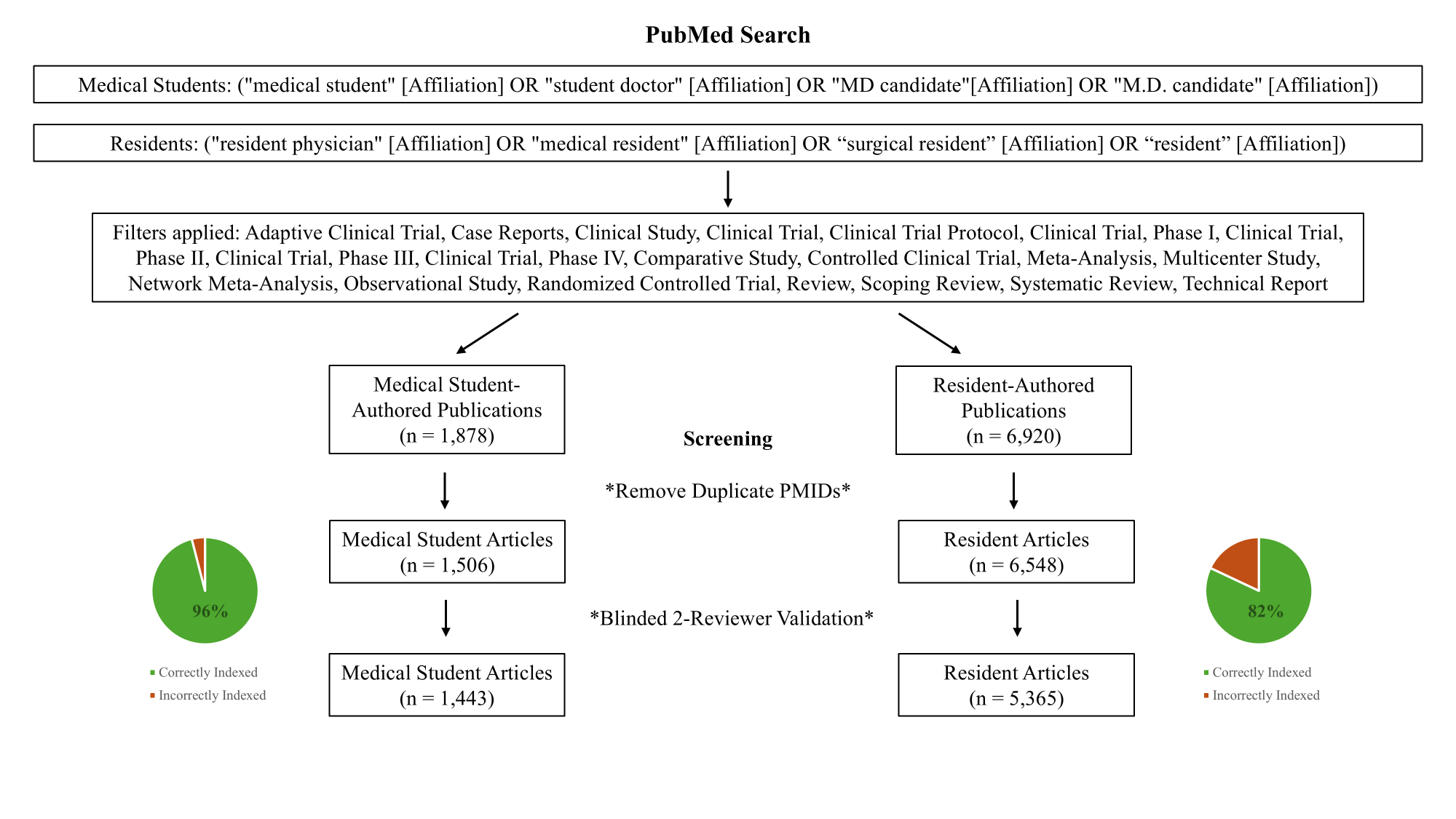

Supplement: S1 Fig — PubMed Search queries for medical student and resident-authored articles. The number of articles identified at each step, filters used, and accuracy of each search strategy is identified to enhance reproducibility. (TIF) [file pone.0343160.s001.TIF]
