# Supplementary material for: Medical student research productivity and scholarly impact: A 20-year bibliometric comparison with medical residents
Source: PLoS One. 2026 Feb 23;21(2):e0343160. doi: 10.1371/journal.pone.0343160 (PMC12928410; doi:10.1371/journal.pone.0343160)
Supplement: Attachment — Submitted filename: PLOS ONE R1 Response to Reviewer Comments.docx [file pone.0343160.s005.docx]

To the Editors and Reviewers at PLOS One,

Thank you for providing this thoughtful and insightful feedback. We have used it to strengthen our study, and we hope that our revised manuscript reflects equal care and prudency. Please see below our responses to each editorial and reviewer's comment. Thank you again for your time and consideration.

| 1. Please ensure that your manuscript meets PLOS ONE's style requirements, including those for file naming. The PLOS ONE style templates can be found at  <https://journals.plos.org/plosone/s/file?id=wjVg/PLOSOne_formatting_sample_main_body.pdf>  and  <https://journals.plos.org/plosone/s/file?id=ba62/PLOSOne_formatting_sample_title_authors_affiliations.pdf> | To our knowledge, the manuscript meets formatting requirements. |
| --- | --- |
| 2. Please include a copy of Table 4 which you refer to in your text on page 14. | Thank you for bringing this to our attention, we intended for this to say Figure 4, not Table 4, which we have now corrected. |
| 3. Please include captions for your Supporting Information files at the end of your manuscript, and update any in-text citations to match accordingly. Please see our Supporting Information guidelines for more information: <http://journals.plos.org/plosone/s/supporting-information>. | Thank you, a caption for each supporting information file (S1-S4) has now been added to the end of the manuscript. |
| 4. If the reviewer comments include a recommendation to cite specific previously published works, please review and evaluate these publications to determine whether they are relevant and should be cited. There is no requirement to cite these works unless the editor has indicated otherwise. | No specific comments were made to cite previously published works. |
| 5. Please review your reference list to ensure that it is complete and correct. If you have cited papers that have been retracted, please include the rationale for doing so in the manuscript text, or remove these references and replace them with relevant current references. Any changes to the reference list should be mentioned in the rebuttal letter that accompanies your revised manuscript. If you need to cite a retracted article, indicate the article’s retracted status in the References list and also include a citation and full reference for the retraction notice. | After review, none of our included references have been retracted. |
| 6. However, while the study includes international data, the dominant cohort and highest-impact publications are from the United States. The findings may not generalize well to medical education systems in other countries with different training structures, research culture, or publication incentives. | Thank you for bringing this to our attention, and we agree that having a dominant US cohort would result in potentially poor generalizability to international medical education systems given various factors. However, we address this in our analysis by including 697 articles from US students and 748 articles from international students, providing nearly even cohorts, with a slightly larger international cohort. We acknowledge that factors such as training structure, research culture, or incentivization may vary from country to country, but in this paper, we focus on doing our best to objectively analyze and compare medical student research the way it stands presently.  Please see Figures 7-8. |
| 7. The authors acknowledge that using affiliation-based PubMed queries to identify medical students and residents is "not comprehensive" and can lead to misclassification | Yes, we agree and stand by the fact that affiliation-based PubMed queries to identify medical students and residents is not without error, and that misclassification may occur. However, we aimed to limit this by applying the method consistently across cohorts and acknowledge the inherent limitations associated with this method. With the greater than 95% accuracy in identifying medical student-authored articles after a blinded, two-reviewer validation process, we believe it remains the best-available approach to collect and analyze this quantity of data in the most accurate way possible.  Please see Figure S1 for additional information. |
| 8. The analysis is limited to English-language articles indexed in PubMed only, which excludes non-English publications and research from databases not captured in PubMed. This significantly limits generalizability to global medical student research; so try to expand database coverage beyond Pubmed | Thank you for highlighting this important consideration. We agree that limiting the analysis to English-language articles indexed in PubMed may exclude relevant non-English publications and studies indexed in other databases, and we have explicitly acknowledged this as a limitation in the revised manuscript. Nonetheless, we were able to capture 750 international medical student-authored articles from over 20 countries.  However, to best progress dialogue in this field, we have also deemphasized findings on differences between U.S. and international medical student cohorts in terms of research quality and instead highlighted the substantial growth in research volume and quality evident across both cohorts over the last two decades.  Please see abstract lines 45-49 and results lines 286-287. |
| 9. The study does not quantify the influence of medical student or resident authors by their authorship position (first author, middle author, corresponding author) or account for the role of the primary investigator. This means we cannot determine whether students were the driving force or minor contributors to the work. Contact corresponding authors or institutions directly to confirm authorship status and role (first author, senior author, mentee vs. mentor-led). This would reduce affiliation-based misclassification and clarify the student's actual contribution level. | Thank you for providing this feedback. We also believe it is relevant to investigate the influence of medical student authorship position on article RCR, and this is an area of active work amongst our team.  We have found that (635/1445) or 44% of indexed articles had medical students as the 1^st^ author and have included this in our manuscript.  Though, given the volume of data that we currently present in this paper, we believe that expanded analysis by authorship position could be better addressed in a future paper focused on aspects of team structure, mentorship, and specific authorship roles.  Lastly, it’s also worth acknowledging that per ICMJE guidelines, all authors – regardless of medical student vs. resident vs. attending status – must have made substantial, meaningful contributions toward the project and manuscript, regardless of specific author order.  Specifically, “the ICMJE recommends that authorship be based on the following 4 criteria: Substantial contributions to the conception or design of the work; or the acquisition, analysis, or interpretation of data for the work; AND Drafting the work or reviewing it critically for important intellectual content; AND Final approval of the version to be published; AND Agreement to be accountable for all aspects of the work in ensuring that questions related to the accuracy or integrity of any part of the work are appropriately investigated and resolved.”  We hope our expanded discussion in the manuscript and our provided rationale adequately address this comment. |
| 10. Present separate analyses for first-author vs. other-author positions. Students as first authors likely drive research conception and execution more substantially than when appearing as co-authors, making this distinction important for interpreting the findings. | Thank you for raising this point. We agree that medical students listed as first authors are likely to contribute more substantially to a study than those listed in non-first-author positions. However, given the scope and number of analyses already presented, we believe that a detailed authorship-position subanalysis is beyond the intended focus of the current manuscript.  Nonetheless, we have identified that nearly half of included medical student publications (635/1445) list a medical student as a 1^st^ author, which indicates that medical students likely drive research conception and execution in a substantial portion of their works. This point has been included in the revised manuscript.  We believe that a more granular evaluation of authorship position, particularly its association with article-level impact metrics such as RCR, would be best addressed in a future, dedicated manuscript with appropriate analytic focus. |
| 11. The paper frequently interprets citation counts and RCR (Research Comparison Rate) directly as evidence of research "quality" or "high-level contribution." However, RCR essentially reflects relative academic influence, not methodological rigor, originality, or research quality itself. Authors need to clearly distinguish between "academic influence" and "research quality" in the abstract, discussion, and conclusion. | Thank you for providing this feedback – we believe that RCR reflects relative academic influence, which is widely seen as a proxy for quality and, broadly, contribution to the literature. Similar to the use of journal impact factors, RCR has been demonstrated to correlate strongly with the opinions of subject matter experts, alluding to its validity as a proxy for both quality and influence. We have provided minor changes in the manuscript to emphasize RCR directly measures influence and, by proxy only, quality.  Hutchins BI, Yuan X, Anderson JM, Santangelo GM. Relative Citation Ratio (RCR): A New Metric That Uses Citation Rates to Measure Influence at the Article Level. PLoS Biol. 2016;14(9):e1002541. Published 2016 Sep 6. doi:10.1371/journal.pbio.1002541 |
| 12. Several core conclusions rely on the analysis results after excluding case reports. However, given the high proportion of case reports in the research of trainees, this exclusion may introduce selection bias and unintentionally inflate the overall impact index. | Thank you for providing this feedback. Figure 2a shows that case reports did account for 30.63% (n=442) of the included medical student articles but had a significantly lower mean RCR than all other article types included (Figure 2b). Resident-authored case reports accounted for 65% (n=3435) of included resident articles. With this in mind, we believe that benchmarking medical students vs. residents with and without case reports allows for a balanced analysis, given the higher proportion among resident authors. Furthermore, we provide the data directly to the audience in hopes that they may interpret the information most pertinent to them and draw their respective conclusions accordingly.  Overall, we have also deemphasized the results excluding case reports in the abstract and discussion, rather focusing on the growing quality of medical student research overall where, in 2023, the median publication RCR was 0.8, which includes case reports. |
| 13. While this paper focuses on the research contributions of medical students, the analysis does not address the impact of authorship order on the paper's contribution. Authorship alone cannot determine an author's actual academic role in the research. | Thank you for this comment. Please see responses to comments 9 and 10, above. |
| 14. Although RCR is a field-normalized indicator, systematic differences still exist between different research types (e.g., reviews, meta-analysis, and original research). The current manuscript has not fully discussed the potential impact of differences in research type composition on RCR results. | Thank you. This is certainly a valid comment based on how RCR was developed, which we also sought to address via analyses by article type (e.g., meta-analysis, review etc.).  Please see analyses in figure 2 and lines 198-214 of the manuscript. |
| 15. Using only PubMed data may introduce subject, language, and regional biases, and the impact on international comparison results requires further discussion. | Thank you, this has been further addressed in the discussion and limitations sections. This is also a point we noticed while preparing this manuscript, where metrics like RCR may not be evenly applied internationally. In addition to the manuscript, we have also launched and included the ImpactLens RCR tool. <https://impactlensgit.netlify.app/>  This tool enables medical student researchers from any institution to share and benchmark their articles via field and location-normalized RCR metrics, to account for regional variability in article visibility that otherwise confounds approximations of research “impact” or “quality.” |
| 16. Some differences (the RCR of papers by US medical students is higher than that of international medical students (0.58>0.40, p = 0.02)) are statistically significant, but not in terms of research ability itself. Their actual educational or policy implications have not been fully explained. | Thank you for this thoughtful comment. We agree that differences in Relative Citation Ratio should not be interpreted as differences in individual research ability. Rather, RCR reflects article-level citation performance, which is influenced by multiple contextual factors beyond author skill, including institutional research infrastructure, funding availability, mentorship density, journal visibility, and citation practices across regions.  To address this, we have revised the results to more explicitly frame this finding as a reflection of structural and systemic differences in research environments rather than inherent differences in medical student research ability.  These findings highlight opportunities for targeted educational and policy interventions – such as international research partnerships, enhanced mentorship structures, and equitable access to research infrastructure – to support high-impact medical student scholarship globally. The conclusions have been revised to reflect this need. |
| 17. The figures and tables in this paper are rich in information but not clear enough, and their correspondence with the core arguments of the text is not direct enough. | Thank you for pointing this out. Tables 1-3 have been revised for clarity, with table 2 now much more succinct. Figures 1,4, and 6 have also been adjusted for clarity. |
